# Laparotomy, with Suture of Intestinal Canal

**Published:** 1885-01-20

**Authors:** J. McF. Gaston

**Affiliations:** Professor of Principles and Practice of Surgery in the Southern Medical College, Atlanta, Georgia


### Southern Medical Record.

EDITORS:

THOMAS S. POWELL, M.D. R. C. WORD, M.D.

*R. C. WORD, M.D., Managing Editor.*

☞ All Communications and Letters on Business connected with the RECORD must be addressed to the Managing Editor.

VOL. XV. ATLANTA, GA., JANUARY 20, 1885. No. 1.

### ORIGINAL AND SELECTED ARTICLES.

### LAPAROTOMY, WITH SUTURE OF INTESTINAL CANAL.

By J. McF. GASTON, M.D.,

Professor of Principles and Practice of Surgery in the SOUTHERN MEDICAL COLLEGE, Atlanta, Georgia.

Being invited by Dr. G. G. Roy to visit Birt. Blanchard, on the afternoon of December 6, 1884, it was stated that on the previous night he had received two pistol shots, and it was found that one had passed through the outer tissues of the left thigh, while the other had entered the abdomen about four inches below the umbilicus, and diverging slightly to the left of the median line.

After an exploration with an elastic bougie, which satisfied us that the ball had penetrated the abdominal cavity, and most probably wounded the coils of intestine in its course, it was thought best to seek relief from the consequences, manifested in tenderness and distention of the abdomen, by an early operation. This being explained to the patient by Dr. Roy, as offering the most favorable prospect for a good result under all the circumstances of his case, the proposition was accepted by him ; and, without further delay, we proceeded with the preliminary measures for opening the abdomen.

The patient being brought under the influence of chloroform, the first step taken was to pass a directory into the aperture made by the ball, and, turning the point upwards as soon as it reached the cavity, this was kept in close contact with the peritoneal lining so as to avoid any portion of the intestinal canal. A superficial incision over the line of the directory was made through the skin and subcutaneous tissue, followed by a dissection through the greater portion of the linea alba, when a probe-pointed bistoury, being introduced at the opening of the ball, was passed along the groove in the directory so as to divide the remaining fibres of the linea alba and the peritoneal membrane up to the umbilicus. Proceeding in a similar manner, the opening was extended an inch below the orifice of the ball, thus affording easy access to the cavity for the examination of the intestines. A coil of the ileum being hooked up with the finger in a direct line with what was supposed to be the course of the bullet, this canal was drawn out several inches before any aperture in its walls was reached, and it gave evidence of a highly engorged condition, if not an inflammatory state, of the serous investment.

The first lesion which was discovered had evidently resulted from the passage of the ball on one side of the canal, leaving a ragged oblong aperture in its wall, which being smoothed by cutting away its margin with scissors, silk suture of the continuous order was used to close the wound. In drawing upon what proved to be the lower segment of the bowels, another lesion was found within a few inches of that which had been closed, having two apertures through the wall from the passage of the ball transversely, leaving a bridge between, which was divided along with the trimming of the ragged edged, and the whole united with a continuous silk suture. In the application, a single stitch, like in the interrupted suture, was knotted at the beginning, and a draw-knot was made at the close, the ends of the thread being cut off at both extremities of the continuous suture in the wound.

Traction upon the bowel in the same direction revealed two other distinct openings, a few inches from each other, which were closed without any trimming of the edges, as there was less irregularity than in the margins of those previously encountered ; and, though the rule laid down for bringing the outer or serous surfaces in juxtaposition when approximating the margins, was recognized as proper, it was not accomplished by either of us very satisfactorily in bringing the edges together with the stitches. While the line of union was secured in such form that the respective serous and mucous membranes came into contact accurately, the classical and technical folding inwards of the serous investment of the bowel was not effected in the several sutures put in by Dr. Roy and myself on this occasion. When a further effort was made to draw out more of the bowel, and it offered resistance, we inferred, from a digital exploration, that the ileo-coecal attachment prevented it, and our attention was then directed to that portion of the canal beyond the point of the first suture. Within two feet of this there were two other orifices made by the passage of the bullet through the wall of the intestine, several inches apart, and closed without trimming. Finding no more openings, upon drawing out a considerable portion of the canal, it was inferred that all the wounded portion had passed under inspection, and the only aperture found in the mesentery gave no signs of bleeding, so that it was returned, without stitching, along with the coils of intestine.

An examination, previously had, revealed the presence of some coagula of blood, mixed with portions of the intestinal contents, which had been partially removed by carrying pieces of old cotton cloth into cavity of the abdomen, and especially into the pelvic region; and now a thorough cleansing was resorted to, by turning the patient from side to side, while all was swabbed out until the cloths were returned without being stained. A bougie was carried through the urethra into the bladder, so as to examine its walls, and afforded strong assurance that no injury had been inflicted upon it at the time of the shooting.

Supposing that nothing was left undone which lay in our power to secure the advantages of such an operation, a drainage table, having a diameter of one-eighth of an inch, with numerous openings in its walls, about eighteen inches long, was so disposed in the lower portion of the cavity as to take up any serous effusion, and the outer extremity was carried out at the upper portion of the abdominal incision.

With the assistance of Dr. W. A. Smith in closing the abdominal incision, the internal peritoneal membrane was brought together by a distinct interrupted suture with fine silk thread, and the external cutaneous investment with the intervening tissues were united with a doubled thread by the interrupted suture separate from that in the lining membrane. Having turned the patient again from side to side, with a view to express any fluid which might remain in the abdominal cavity, the end of the tube was secured by a strong silk thread around the body, and a large compress of soft cotton was bound over the incision by a broad belt of cloth. The depressed state of our patient became a matter of concern. The chloroform had been suspended for some time, and there was consciousness to the pain of passing the needle in the skin for the last suture; but the shock from the bowels being exposed for so long a time, or from the number of the sutures in the intestinal canal, was attended with such prostration that Dr. Roy proceeded to use subcutaneous injections of brandy and of sulphuric ether, with an enema of whisky, before reaction was established. Bottles with hot water were also ordered to be kept to his lower extremities, with friction, to arouse the circulation and heat in his body. Eventually consciousness returned, and the natural warmth was restored, with improvement in the pulse, so that Dr. Smith, who had undertaken to watch the case, considered his condition so favorable as to enable him to withdraw from his bedside before midnight.

After passing the rest of the night in compative comfort, and having two evacuations from his bowels without any admixture of blood, he took some chicken soup and other light nourishment towards morning ; but about sun rise there were indications of a return of prostration, which rapidly increased until he sank in death at 9 o'clock in the morning.

A post mortem, at 3 o'clock in the afternoon, gave evidence of peritonitis throughout the abdominal cavity, and a careful examination of the intestinal tract led to the discovery of two additional openings in that portion near the ileo-coecal attachment, from which, however, there was no secondary effusion of blood, and very little escape of matter from the bowel, so that the oversight in not securing these openings at the time of the operation had nothing to do with the fatal result.

After diligent search, by Drs. Smith and Parks, the ball could not be found, it having impinged upon the body of one of the lumbar vertebra and glanced, so that its course could not be traced.

It is evident, from the time which elapsed from the hour of the accident until that of the operation, on the following afternoon, that peritonitis had already been set up in the cavity of the abdomen ; and hence the prospect of a favorable result was much less than it would have been in an immediate operation. But with nine bullet holes cutting through different sections of the ileum, including from those within two and three inches of the ileo-secal valve to that most remote in its walls—an extent of some ten feet of the intestinal canal—there could have been no very certain calculation upon a happy issue of the operation performed at the earliest practicable period after the accident, even if all the orifices had been discovered and closed on the occasion.

Our failure to detect the injury inflicted at two points near the ileo-coecal attachment was due to the concealment of this portion within the line of the abdominal incision, so that these openings did not appear in the protruding coils of intestine, and there was no reason to suspect the perforation of this part of the bowel which was bound down to the right side at the posterior and lower part of the abdomen, which occurred, doubtless, in the glancing of the ball from the body of the vertebra. In this respect, the post mortem was instructive, and yet the information is not likely to be available in similar cases, as no two accidents involving gun-shot wounds of the intestines bear a very close resemblance to each other in the course of a bullet after becoming turned from a right line by any obstacle.

A comparison of the time-serving and masterly-inactivity proceeding of former times with the results of active interference latterly in perforations of the intestinal canal by bullets from pistols or rifles, offers great encouragement for timely resort to laparotomy and suture of the openings. We have a report of the case recently operated on by Dr. W. T. Bull, at the New York Hospital, in which sutures were made in seven intestinal bullet perforations of the intestine and ball found in the sigmoid flexure of the colon, which illustrates the importance of an early operation, and all must perceive that delay leads to inflammation of the pritoneum—which precludes the successful termination of any operative procedure involving the opening of the abdominal cavity.

As in ovarian cysts, it is found extremely precarious to allow of the escape of their fluid contents into the peritoneal sac; so the diffusion of matters from the intestinal canal amongst the coils of the bowels and the folds of the mesentery are prone to give trouble. In either case, there is great difficulty in cleansing the cavity with sponges or cloths, and more or less irritation from friction with these mops, and it has occurred to me that a more salutary result might be secured from flooding the entire abdominal cavity with tepid water, and passing it amongst the folds of the intestines and the mesentery by a syringe, so as to detach any particles of foreign effete matter, and cause them to pass away in solution with the overflow repeatedly of the pritoneal receptacle. It is known that the utmost distention from serous effusion in ascites causes no great inconvenience, and in like manner every crevice could be washed out by a free use of simple or medicated water.

---

**Paste for Comedones.**—Dr. A. Van Harlingen recommended at the last meeting of the American Dermatological Association, the following formula for the removal of comedones (acne); it was first suggested by Unna: Glycerine, 3 parts; vinegar, 2 parts; kaolin, 4 parts.—*Medical Age.*

